# Growth kinetics of endosymbiont *Wolbachia* in the common bed bug, *Cimex lectularius*

**DOI:** 10.1038/s41598-018-29682-2

**Published:** 2018-07-30

**Authors:** Michael L. Fisher, David W. Watson, Jason A. Osborne, Hiroyuki Mochizuki, Matthew Breen, Coby Schal

**Affiliations:** 1 Department of Entomology and Plant Pathology, North Carolina State University, Raleigh, North Carolina USA; 2 United States Navy Medical Service Corps, Raleigh, USA; 3 W.M. Keck Center for Behavioral Biology, North Carolina State University, Raleigh, North Carolina USA; 4 Department of Statistics, North Carolina State University, Raleigh, North Carolina USA; 5 Department of Molecular Biomedical Sciences, North Carolina State University, Raleigh, North Carolina USA; 6 Center for Human Health and the Environment, North Carolina State University, Raleigh, North Carolina USA

## Abstract

The common bed bug, *Cimex lectularius* harbors the endosymbiotic microorganism, *Wolbachia* (*w*Cle), in a gonad-associated bacteriome as an obligate nutritional mutualist. The obligatory nature of this association suggests that all individuals in *C. lectularius* populations would be infected with *w*Cle. However, studies spanning the past several decades have reported variation in both infection frequency and relative abundance of *w*Cle in field-collected samples of bed bugs. Since the growth kinetics of *w*Cle is poorly understood, the objective of this study was to quantify *w*Cle over the life cycle of two strains of *C. lectularius*. Our results highlight that *w*Cle is dynamic during bed bug development, changing relative to life stage, intermolt stage, and blood-fed status. These results suggest new hypotheses about the coordination of *Wolbachia* growth and regression with its host’s physiology and endocrine events. The observed quantitative modulation of *w*Cle during the bed bug life cycle and during periods of starvation may explain the disparities in *w*Cle infections reported in field-collected *C. lectularius*.

## Introduction

Insect bodies are inhabited by diverse bacterial communities, ranging from parasitic, commensal, to facultative or obligatory mutualistic associations with their host^1–3^. Bacteria are typically harbored within the insect gut and as intracellular associates. The gut microbiome often plays a role in nutrition, development, defense or communication, and may consist of highly complex and somewhat varied communities of bacterial species^1,4,5^. Gut microbiomes are generally horizontally transmitted and insects have evolved specialized strategies, such as coprophagy and proctodeal feeding, to acquire the proper microbes^4^. Several transovarially transmitted, obligate intracellular symbionts occur in insects that subsist on nutritionally poor or unbalanced diets; among these are *Blattabacterium* spp. in cockroaches, *Buchnera* in aphids, *Portiera* in whiteflies, *Baumannia* in leafhoppers, *Wigglesworthia* in tsetse flies, and *Wolbachia* in a wide range of insect lineages including bed bugs^1,2,6,7^. Interestingly, these bacteria are transmitted with high fidelity within lineages, so that all progeny of a carrier mother are infected. These “heritable” symbionts vary both in their contributions to their hosts and in their localization within the host, some residing systemically in various tissues, some localize diffusely in cells associated with the fat body or integument (mycetocytes) or specialized structures, bacteriomes (or mycetome), often associated with the gonads^1,2,6,7^.

Bed bugs are obligatory hematophagous insects with hemimetablous development from egg to adult through five nymphal stages (instars), each of which requires a blood-meal to molt to the next stage. The common bed bug, *Cimex lectularius* (Cimicidae) harbors a hereditary, Gram (-), intracellular *α*-proteobacterium, *Wolbachia* that is primarily concentrated in a gonad-associated bacteriome. *Wolbachia*’s relationship with *C. lectularius* presumably evolved from a facultative association to obligate mutualism where the bacteria garner protection and nutrients within their host in exchange for supplementing the host’s nutritional needs^8–10^. The *C. lectularius* strain of *Wolbachia* (*w*Cle) belongs in the F supergroup^11^, and its genome was found to be similar to other insect-associated facultative *Wolbachia* strains in the A, B and D supergroups, but with the exception of the presence of genes encoding complete biotin (vitamin B_7_) and riboflavin (vitamin B_2_) biosynthetic pathways^9,10^. Bed bugs cured of *w*Cle with antibiotics are less fit, have poor adult emergence and severely reduced egg hatch rates, but recover significantly with B vitamin supplementation^9,10^.

The obligate association of *w*Cle with its host, transovarial transmission, and poor performance of bed bugs cured of *w*Cle infection, all would suggest that all *C. lectularius* populations worldwide should be infected with *w*Cle. Yet, early research^12,13^ and more recent studies^14–17^ reported variation in both infection frequency and the relative abundance of *w*Cle in field-collected samples of bed bugs. While it is possible that some *C. lectularius* lineages have developed facultative associations with *Wolbachia*, these observations could also be a consequence of the intimate synchronization of the respective physiologies of the host and its symbiont. For example, *w*Cle might experience proliferation-regression cycles in relation to the bed bug ingesting and processing a blood-meal, or in relation to bed bug molt and developmental stages.

The growth kinetics of *w*Cle in *C. lectularius* is poorly understood, but endosymbiont titers have been quantified in other organisms in relation to host development. For example, *Wolbachia* fluctuates over the course of the life cycle of its host, the nematode *Brugia malayi*. The endosymbiont resides intracellularly inside host-derived vacuoles in the hypodermal cord cells^18^, and the number of *Wolbachia* per nematode cell nucleus remains low in second (L2) and third (L3) stage larvae, but increases significantly with rapid multiplication in fourth stage larvae (L4), likely related to oogenesis^18,19^. The titer of the primary endosymbiont *Rhodococcus rhodnii* fluctuates over time in the gut of *Rhodnius prolixus*, a blood-feeding insect closely related to bed bugs. Gut titers are highest ~5 days following ingestion of a blood-meal, reaching as high as 10^8^ colony-forming units per milliliter in the hindgut, but gradually decline over time^5,20^. Third, 4^th^, and 5^th^ instar *R. prolixus* nymphs also have more *R. rhodnii* than 1^st^ or 2^nd^ instars^21^. Copy number of the *rrs* gene that codes for 16S rRNA in the aphid endosymbiont *Buchnera*, increases in relation to aphid mass over the course of aphid development^22^, and *Buchnera* declines with host age^3^.

Nonetheless, to our knowledge, no reports have quantified the endosymbiont titer within the intermolt stage and in relation to feeding in bed bugs. We were particularly interested in this relationship as well as the *w*Cle titer before and after the nymphal and adult molts. Moreover, we sought to understand whether the reported highly variable relative abundance of *w*Cle in field-collected *C. lectularius* could be attributed to variation in bed bug developmental and blood-fed status. In this study, we quantified the amount of *w*Cle during nymphal and adult development in two strains of *C. lectulariu*s using droplet digital (ddPCR), which has greater accuracy of quantification at low target concentrations than qPCR, and absolute quantification does not require reference genes or the generation of a standard curve^23–25^. The 16S rDNA gene of *Wolbachia* has been reported as a single copy in the two supergroup A strains *w*Mel and *w*Ri in *Drosophila melanogaster* and *Drosophila simulans* respectively^26^, in the supergroup B strain *w*Pip in the mosquito *Culex quinquefasciatus*^27^, and in the supergroup D strain *w*Bm in *Brugia malayi*^28^. Similar to *Wolbachia* in A, B, and D supergroups, 16S copy number for *w*Cle exists as a single copy (Ribosomal Database Project, University of Michigan, https://rrndb.umms.med.umich.edu/). Our results demonstrate that the relative abundance of the *w*Cle endosymbiont fluctuates dramatically over the life cycle of *C. lectularius* and in relation to its blood-fed status. These results may also explain, at least in part, the high variation in infection frequency in field-collected bed bugs.

## Results

**Figure 1:**
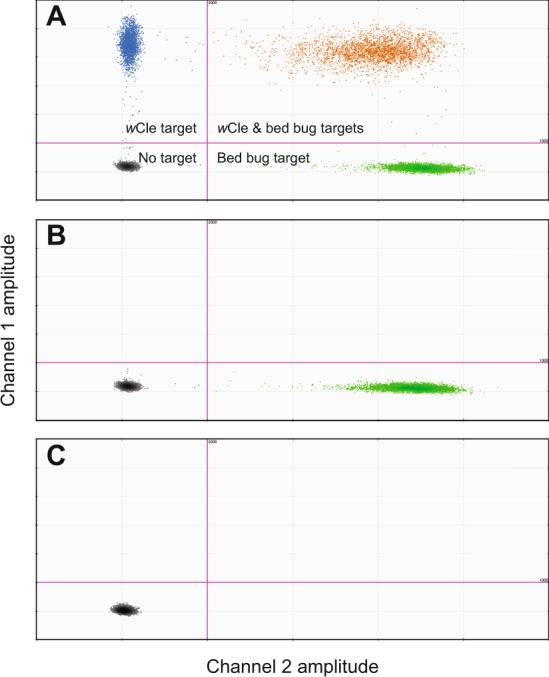
Droplet digital PCR optimization results. Copy number/µl of DNA for *Wolbachia* and *Cimex lectularius* (**A**), *Wolbachia*-free *Cimex lectularius* removed from antibiotics for 90 d maintained on blood fortified with vitamins only (**B**), and no-template control (**C**). *Wolbachia* (*w*Cle) droplet spectrum (blue), *Cimex lectularius* droplet spectrum (green), droplets with both targets (orange), and droplets with neither target (gray).

We designed a ddPCR duplex assay for the absolute quantification of target amplicon copy number in bed bugs and their associated *Wolbachia*. The ddPCR optimization resulted in little variation in samples, where coefficients of variation of estimated DNA copy numbers of bed bug and *Wolbachia* in the five control samples were 2.1% and 2.4% respectively, with a mean (±SE) of 632,480 ± 3,565 for bed bug DNA (RPL18) per individual, and 385,920 ± 4,473 for *Wolbachia* DNA (16S rRNA) per individual (Fig. 1A). The *Wolbachia*-free control bed bugs removed from antibiotics 90 d post-antibiotic treatment contained 532,480 ± 7,634 DNA copies of RPL18 and no detectable *Wolbachia* DNA (Fig. 1B), and no DNA was detected in the no-template controls (Fig. 1C). However, one Harold Harlan (HH) bed bug and five Jersey City (JC) bed bugs were excluded from further analysis because they contained <15 copies/µl of the bed bug RPL18 or *Wolbachia* 16S rRNA genes.

**Figure 2:**
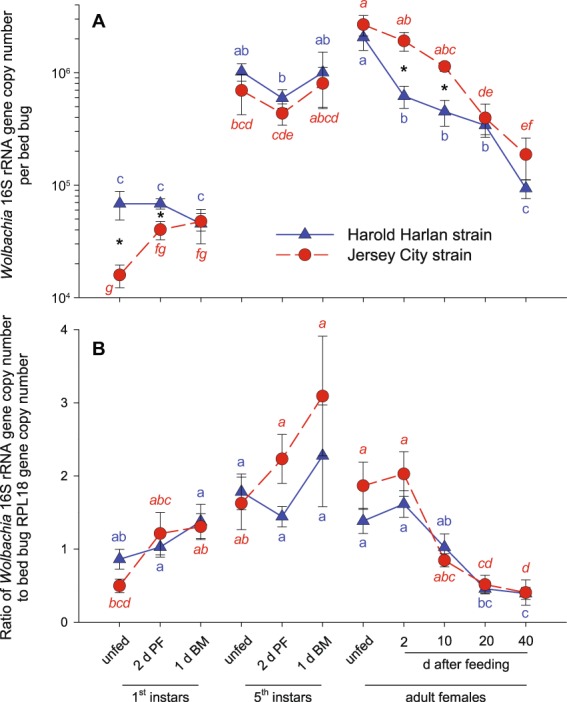
Relationship of *Wolbachia* and bed bug DNA across development of the Harold Harlan (HH) and Jersey City (JC) strains of *Cimex lectularius*. *Wolbachia* 16S rRNA amplicon copy number (mean ± SE) per bed bug (**A**; note logarithmic scale), and the ratio of *Wolbachia* 16S to RPL18 reference gene per bed bug (**B**). For each nymphal stage (1^st^ and 5^th^), represented are unfed newly molted nymphs, 2 days post-feeding (2 d PF), and 1 day before the molt to the next stage (1 d BM). For adult females, days after a blood-meal are shown. Each mean represents *n* = 7–9 individual bed bugs. Color-coded letters correspond to the respective strain of *Cimex lectularius* (JC also in italics) and means sharing the same letter (and the same color) are not significantly different (*p* > 0.05) within each strain (ANOVA and Tukey’s HSD on log10-transformed values, implemented in SAS). Asterisks denote significant differences (**p < 0.01) between respective means of the two strains at the same physiological stage.

*Wolbachia* titer per bed bug ranged from 45,428 ± 15,349 in 1^st^ instars 1 d before the molt to 2,063,796 ± 484,523 in newly emerged unfed adult females in the HH strain, and from 15,865 ± 3,615 in unfed 1^st^ instars to 2,672,853 ± 551,627 in newly emerged unfed adults in the JC strain. Adults and late 5^th^ (last) instars had the greatest variation in *Wolbachia* per bed bug (Fig. 2). Since there was evidence of strain*stage interaction (*F* = 3.69; df = 10, 170; *p* = 0.0002), the strain means were compared separately at each stage, with the strain differences significant at only three stages (10d PF Adult, 2d PF Adult, and Unfed 1^st^). However, neither the strain nor strain*stage interactions were significant for the ratio of *Wolbachia* DNA to bed bug DNA. None of the comparisons of strains at each fixed stage were significant for the ratio (Fig. 2B).

### First instars

**Figure 3:**
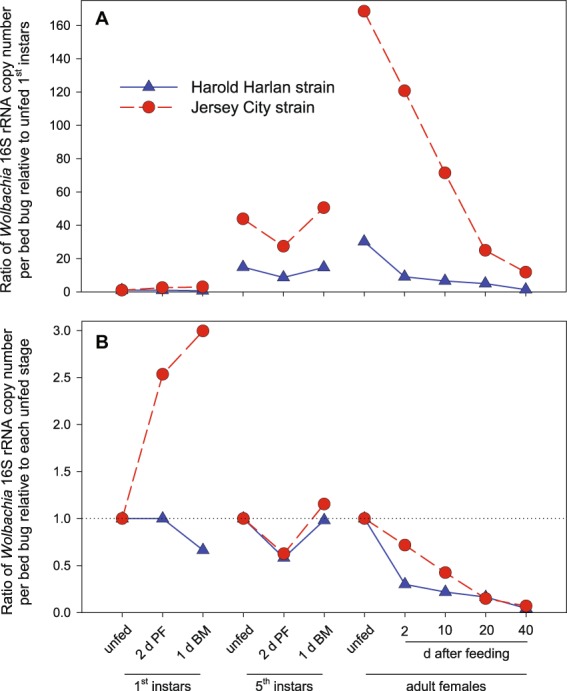
Relationship of *Wolbachia* and bed bug DNA across development of the Harold Harlan (HH) and Jersey City (JC) strains of *Cimex lectularius*. The ratio of *Wolbachia* 16S rRNA amplicon copy number per bed bug across development relative to unfed bed bugs of each of the three life stages. For each nymphal stage (1^st^ and 5^th^), represented are unfed newly molted nymphs, 2 days post-feeding (2 d PF), and 1 day before the molt to the next stage (1 d BM). For adult females, days after a blood-meal are shown. Each mean represents *n* = 7–9 individual bed bugs.

*Wolbachia* titer per bed bug in the HH strain remained low ($\bar{X}$ = 60,449) and steady in 1^st^ instar bed bug 2 d after feeding, but declined just before the molt to 2^nd^ instar (Fig. 2A); nevertheless, the titer was not significantly different among the three stages of 1^st^ instars. In contrast, in the JC strain *Wolbachia* titer significantly increased 3-fold from the start to the end of the 1^st^ instar (Fig. 2A). This difference between the two strains is better illustrated in Fig. 3, where *Wolbachia* titer is normalized relative to the respective titer in unfed 1^st^ instars. Significant differences between the two strains were evident in unfed 1^st^ instars and 2 d after feeding (Fig. 2A). However, the ratio of *Wolbachia* DNA to bed bug DNA, which normalizes for bed bug stage and size, increased monotonically in both strains during the 1^st^ instar with no significant differences between them (Fig. 2B).

### Fifth instar

In both bed bug strains there was a large and significant increase in *Wolbachia* titer between the end of the 1^st^ instar and the start of the 5^th^ instar, ~22-fold increase in HH bugs and ~15-fold increase in JC bugs (Fig. 2A). In both strains, a decline (~42% in HH, ~37% in JC) in blood-fed bugs was followed by a slight increase in *Wolbachia* per bed bug just before the adult molt. However, the three stages of HH 5^th^ instars did not differ significantly, and neither did the JC 5^th^ instars (Fig. 2A). The ratio of *Wolbachia* DNA to bed bug DNA continued to increase through the end of the 5^th^ instar (Fig. 2B), indicating 1.3–1.9-fold faster proliferation of *Wolbachia* DNA than host DNA during this period in both bed bug strains.

### Adult females

Another large, but not statistically significant, increase in *Wolbachia* per bed bug occurred within one day between the last instar and the adult molt (~2-fold increase in HH bugs, ~3.3-fold increase in JC bugs) (Fig. 2A). However, this increase disappeared when *Wolbachia* DNA was normalized to the amount of bed bug DNA (Fig. 2B), suggesting that increases occurred in both *Wolbachia* and body mass. Subsequently, in the adult stage in both strains, *Wolbachia* titer declined significantly with prolonged starvation even when *Wolbachia* DNA was normalized relative to bed bug DNA. *Wolbachia* titer per HH bed bug declined by 95.5% 40 d after feeding, and in JC this decline was ~93.0% (Fig. 2A).

We generated a normalization within each life stage, relative to unfed 1^st^ instars, unfed 5^th^ instars and unfed adult females (Fig. 3). As already mentioned above, the patterns during the 1^st^ instar diverged dramatically between the HH and JC strain, with a 3-fold increase in *Wolbachia* in JC bed bugs and a 34% decline in HH bed bugs from the start to the end of the 1^st^ instar. The *Wolbachia* patterns in the 5^th^ instar and adult stage were similar for both strains when the absolute *Wolbachia* DNA titer per bed bug was normalized to the beginning of each developmental stage (Fig. 3).

## Discussion

Our results highlight that *Wolbachia* of *C. lectularius* (*w*Cle) is dynamic during host development, changing relative to life stage, intermolt stage, and blood-fed status. Overall, we observed that (a) Neonate unfed, lab-reared, bed bugs (HH strain) had 4.3-fold more *w*Cle per bug than field-collected bugs (JC strain), and when normalized to the amount of bed bug DNA a 1.7-fold difference remained; (b) *w*Cle DNA titer per bed bug increased ~30-fold (HH) and ~168-fold (JC) between the 1^st^ instar and adult stages; (c) The largest increase (~15-fold HH, ~44-fold JC) was between the 1^st^ and 5^th^ instars; (d) *w*Cle DNA titer increased throughout both 1^st^ and 5^th^ instars relative to bed bug DNA titer; (e) In both 5^th^ instars and adult females, *w*Cle levels declined significantly within 2 d of ingesting a blood-meal; (f) In adults, *w*Cle decreased by ~95% (HH) and ~93% (JC) after 40 d of starvation to titers approaching those observed in 1^st^ instars; and (g) Adults of the JC field-collected strain accumulated more *w*Cle per bug, and also relative to the amount of bed bug DNA, than the lab-maintained HH strain, and they retained more *w*Cle through starvation. We also conclude that ddPCR is highly sensitive and therefore appropriate for quantifying absolute amounts of *Wolbachia* in bed bugs as well as *Wolbachia* DNA relative to bed bug DNA. Finally, the observed modulation of the amount of *w*Cle during the bed bug life cycle and during periods of starvation may explain the disparities in *w*Cle infections reported in field-collected *C. lectularius*.

Endosymbiotic bacteria, such as *Wolbachia*, are broadly associated with many insect species in a variety of parasitic, commensal and mutualistic associations^1,2^. As diverse is *Wolbachia*’s location within the host, ranging from a broad systemic distribution, to specific tissues such as fat body cells, or specialized and highly localized bacteriomes^1,2,6,7^. In parasitic associations, particularly those involving reproductive manipulation of the host, *Wolbachia* is expected to be systemically distributed, in relatively low abundance, and its titer in the host to spike during the reproductive stage, in preparation for *Wolbachia*’s transovarial transmission. In contrast, for obligate mutualisms, *Wolbachia* is required for proper growth and development and is therefore expected to be localized in specific tissues, and maintain relatively high populations that provision nutrients to all life stages of its host^8,9,29^.

A central feature of the association of *Wolbachia* with *C. lectularius* is their obligate mutualism, where *w*Cle provisions the bed bug with B vitamins in exchange for being hosted and transmitted as a “hereditary” component of the oocyte. *w*Cle are therefore essential for nutrient synthesis and embryonic development. *w*Cle-cured bed bugs grow more slowly and experience lower fecundity, but these effects can be reversed with biotin (vitamin B7)^8,9^ and riboflavin (vitamin B2)^10^ supplementation of normal blood. This is similar to *Wolbachia* in supergroups C and D, which are associated with filarial nematodes, do not manipulate reproduction of the host, and have obligatory associations with their host, and where nematode fitness is compromised when the *Wolbachia*-association is disrupted^18,19^. Interestingly, *w*Cle belongs to supergroup F, which contains *Wolbachia* strains that associate with both insects and filarial nematodes^11^. It is likely that because of *w*Cle’s nutritional contribution to the bed bug, its titer increases in relation to bed bug growth and development, as we observed. The intimate association of the bacteriome with the gonads likely drives *w*Cle population dynamics.

The blood-meal is the only external source of nutrients for bed bugs, and indirectly for *w*Cle. In contrast to other hematophagous insects such as adult fleas or lice that live exclusively on their host, bed bugs take larger and infrequent blood-meals, and substantial degradation of erythrocytes is delayed up to 12 hours^30^. Nearly 50% of the blood-meal (water weight) is excreted in fecal spots in the first 5 hours post ingestion^31^. Within just 2 d after 5^th^ instar and adult bed bugs ingested blood, the amount of *w*Cle decreased relative to the respective unfed stage (Fig. 2A). It is important to note, however, that relative to the amount of bed bug DNA, *w*Cle continues to increase throughout the 5^th^ instar (Fig. 2B). Whereas the pattern of *w*Cle per 5^th^ instar bug could suggest that *w*Cle might be responding to diminishing energy resources after the blood meal is digested, two lines of evidence argue to the contrary. First, *w*Cle rebounds late in the same instar in the absence of feeding, and second, *w*Cle titer increases throughout the 5^th^ instar in relation to *Cimex* DNA titer. This pattern suggests fine coordination between the physiology of the bed bug and *w*Cle; when blood is available both host and symbiont grow.

In adults, the combined effects of starvation and reproduction also affect *w*Cle populations. As in 5^th^ instars, the ratio of *w*Cle DNA to bed bug DNA increased in adults after the blood-meal, but in adults *w*Cle continued to decrease with prolonged starvation to extremely low levels. Although these females were unmated, *C. lectularius* virgin females oviposit some infertile eggs^32^ and *w*Cle was provisioned to oocytes and lost with oviposited eggs. In the absence of re-feeding, the *w*Cle titer continued to decline. We suspect that if females were offered a blood meal >5 d after their last feeding, and mated to stimulate oocyte development and greater oviposition, *w*Cle numbers would dramatically increase. But this speculation would need to be tested empirically.

The fine coordination of *w*Cle titer with bed bug feeding needs further investigation. *w*Cle appears to respond to nutritional conditions in its host much faster than the time resolution of our study. It is possible, for example, that we missed peaks in *w*Cle populations immediately after the blood meal that damped out within 2 d after the blood meal was ingested. Highlighting this response is *w*Cle’s rapid increase (2-fold in HH, 3.3-fold in JC) in one day between the late 5^th^ instar and the newly emerged adult (Fig. 2A). Notably, since bed bug DNA increased during the molt, the ratio of *w*Cle to bed bug DNA in fact declined in the transition from nymph to adult (Fig. 2B).

Precise mechanisms of coordination of insect-bacterial symbiosis are not well known. Buchner^33^ concluded that in each host-symbiont example he examined, the host was the ultimate regulator of the symbiosis; binary fission occurred at much lower rates *in vivo* compared to related free-living bacteria (*in vitro*). In aphids and leafhoppers, lysosomal activity within mycetocytes selectively breaks down certain symbionts^34,35^, and Hinde^36^ believed that in aphids, the selectivity removed nonviable individuals or was the primary means of insoluble nutrient acquisition from symbionts. Chang and Musgrave^37^ also discovered lysis of symbionts via autophagic vacuoles within the mycetome they termed ‘cytolysomes’ in *C. lectularius*, which suggested a host mechanism of symbiont suppression.

One could postulate that *w*Cle has relinquished control over replication and cell division to its bed bug host through lateral gene transfer or genome reduction, since this symbiosis is a highly specialized obligate mutualism. Interestingly, this does not appear the case. In contrast to other *Wolbachia* genomes, *w*Cle has not undergone extensive genome reduction, or experienced significant loss of genes that control cell division, replication, or are responsible for other essential functions^9^.

The coordination of *w*Cle with host development and physiology may be driven by nutrition or the bed bug’s endocrine cycle. *Wolbachia*, like other intracellular endosymbionts such as *Spiroplasma*, require macronutrients from the host for replication and proliferation^38–40^. Starvation reduced and eliminated *Wolbachia* in the predatory mite *Metaseiulus occidentalis*^41^. Dietary intake strongly influences *Wolbachia* titer in the female *Drosophila* germline; a diet high in sucrose increased *Wolbachia* oocyte titer, but a high yeast diet decreased *Wolbachia* titer in oocytes^42^. Additionally, the ratio of protein to carbohydrate intake modulated *Wolbachia* abundance in *Drosophila*^43^. Glucose metabolism and glycogen storage in *B. malayi* are linked with *Wolbachia* fitness in a metabolic co-dependency pathway shared between the bacteria and its nematode host^44^. In the *B. malayi* system, *w*Bm lacked genes for 2 glycolytic enzymes, 6-phosphofructokinase and pyruvate kinase, and were unable to convert glucose into pyruvate^44^.

Competition between *Wolbachia* infections and the host for nutrients has also recently been suggested. *Wolbachia* had a direct impact on cholesterol availability in *Aedes aegypti* mosquitoes^45^; *Wolbachia*-infected mosquitoes had ~25% less cholesterol than uninfected. Fallon *et al*.^46^ reported that depletion of host cell riboflavin reduced *Wolbachia* infection in cultured mosquito cells, suggesting that *Wolbachia* responded to the availability of riboflavin. In the case of *w*Cle, the bed bug host may out-compete *w*Cle for carbohydrates, lipids, or proteins, and hence offer an explanation to the substantial decline in *w*Cle we observed in starved adult females.

In the related hemipteran *R. prolixus*, a blood-meal stimulates a molt cycle through humoral factors and neuronal signals generated by stretch receptors in the gut^47^. Molting in most insects is initiated by the corpora cardiaca release of prothoracicotropic hormone, which stimulates the prothoracic gland to produce ecdysone. In concert with a low juvenile hormone titer in last instars, the adult molt ensues. Ecdysone is a strong candidate for coordinating host and symbiont physiology. For example, it promotes growth, maturation and sexual differentiation in flagellate protozoans that reside in the hindgut of the wood-feeding cockroach *Cryptocercus*. Thus, when ecdysone production was suppressed in the cockroach, gametogenesis of the protozoan *Trichonympha* ceased within 2 hours, and death occurred after 6–10 hours^48^. Conversely, ecdysone injections stimulated *Trichonympha* gametogenesis in cockroach adults and nymphs^49^. Similarly, juvenile hormone affected intracellular symbionts in the cockroach *Periplaneta americana*^50^. Bacterial symbionts in fat body mycetocytes of four species of cockroaches responded to changes in cockroach life stage and oocyte development and were affected by hormones from the corpora cardiaca^51^. In bed bugs too, *w*Cle may be responding to fluctuations in hormone titers, and proliferate just before the molt.

The results presented in this study suggest new hypotheses about the coordination of *Wolbachia* growth and regression with its host’s physiology and endocrine events. Future experiments could include quantitative measurements of *Wolbachia*’s rapid response to feeding, molting, mating, and oviposition, as well as to manipulations of juvenile hormone and ecdysone titers in the bed bug. As well, the broad-scale changes in *w*Cle in various life stages of *C. lectularius* bear on recent failures to detect *w*Cle in some field-collected bed bugs. This appears to be in conflict with the ostensible obligate symbiosis of *w*Cle and *C. lectularius*, suggesting (a) that *w*Cle titers in some field-collected bugs were below the detection limit of the assays, or (b) that *C. lectularius* lineages may vary in their degree of dependence on *w*Cle. Regarding the latter, the field-collected JC strain had much higher titers of *w*Cle than the lab-adapted HH strain, suggesting that the lab bed bugs might have adapted to frequent and possibly higher quality blood meals by depending less on *w*Cle. Consistent with this suggestion, multiple recent surveys indicate that sizeable human populations in developed countries fail to consume the minimum recommended quantity of B-vitamins^52^; bed bugs feeding on B-deficient people might depend more on their *Wolbachia* associates to provision them with B-vitamins. Conversely, the lab-adapted bugs that fed for many generations on the blood of B-vitamin supplemented well-fed rabbits, possibly became less dependent on *Wolbachia*. Importantly, the *w*Cle-*Cimex* nutritional symbiosis has yet to be investigated with natural field populations.

## Methods

### Insects

Two *Cimex lectularius* bed bug strains were used in these experiments. The Harold Harlan strain (HH) was collected in 1973 in Ft. Dix, NJ, USA. It is an insecticide-susceptible strain used as a standard in many laboratories working on bed bugs. This strain has been maintained at NC State University since 2008 on defibrinated rabbit blood (Hemostat Laboratories, Dixon, CA). The Jersey City (JC) strain of *C. lectularius* was collected in 2008, also in NJ, and maintained on defibrinated rabbit blood at NC State University since then. It is known to be highly resistant to pyrethroid insecticides^53,54^. Both strains were reared at 27 ± 1 °C (Thermo Scientific, Precision model#3727, Waltham, MA, USA) under a photocycle of 12/12 (light/dark). Bed bugs were maintained in small, round plastic containers (5.4 cm diameter × 4.8 cm) that contained pleated paper shelters which contacted plankton netting (0.3 mm mesh) on the top of the container through which bed bugs could feed on blood. Blood was placed in a water-jacketed custom-made glass feeder and warmed to 38 °C with a thermal circulator, as previously described^55^.

### Experimental design

Our objective was to determine if the relative abundance of *Wolbachia* fluctuated during the developmental cycle and in relation with the blood-fed status of *C. lectularius* for both HH and JC strains.

#### Nymphal development

Neonate unfed 1^st^ instars were placed into screen-capped 7 ml glass vials (6–12 nymphs per vial, three replicates) with pleated manila card stock strips for shelter. Bed bugs were fed defibrinated rabbit blood and maintained under the same conditions as described above for colony maintenance. To synchronize the manipulations for this experiment, one group of bed bugs was used as sentinels to predict molting events for the test group. These sentinel groups consisted of five 1^st^ and five 5^th^ instars held in separate vials. They were used to estimate the time interval between feeding and 1 d before the next molt. Bed bugs in these vials were fed the same batch of blood 3 d prior to commencing the experiment. The 1^st^ instar is the first to modulate maternally transmitted *Wolbachia*, whereas the 5^th^ (last) instar prepares for the adult molt. Stages between these were expected, in principal, to be qualitatively similar to the 1^st^ instar.

Three individuals from each vial (*n* = 9) were randomly collected for testing at the following time intervals: unfed 1^st^ instars (not fed for ~3–5 d since hatching), 1^st^ instars 2 d post-feeding, and 1^st^ instars 1 d before molting to 2^nd^ instar. Each bed bug was separately placed into a 1.5 ml microcentrifuge tube with 95% ethyl alcohol (EtOH). Samples were stored at −20 °C until DNA extraction. The same procedure was repeated using newly molted 5^th^ instar females, which were sampled as follows: unfed 5^th^ instars (not fed for ~3–5 d since molting to 5^th^ instar), 5^th^ instars 2 d post-feeding, and 5^th^ instars 1 d before molting to adult.

#### Adult development

Sixty newly molted unfed 5^th^ instar females were placed into 7 ml screen-capped vials with pleated manila card stock, as above. These nymphs were fed to repletion and maintained in the same incubator as other nymphs and under the same conditions. Newly emerged unfed adults were placed in 7 ml screen-capped glass vials (12 females per vial, three replicates), as above, with pleated paper for shelter. Adult females are of particular interest because they provision oocytes with *Wolbachia*. Three unfed females from each vial (*n* = 9) were collected before feeding, the rest of the adult females were fed defibrinated rabbit blood to repletion, and three individuals from each vial (*n* = 9) were collected on the following days after feeding: 2, 10, 20, and 40 d. Collected females were placed individually into separate 1.5 ml microcentrifuge tubes in 95% EtOH and stored at −20 °C until DNA extraction.

### DNA extraction

Total genomic DNA was extracted using the DNeasy Blood and Tissue kit (QIAGEN, Germantown, MD, USA) using a modified protocol for purification of total DNA from animal tissues (spin-column). Heads were removed from 5^th^ instars and adults to reduce interference from eye pigments with fluorescence reading in ddPCR, and individual bed bugs were placed in 1.5 ml microcentrifuge tubes with 180 µl of ATL buffer solution and homogenized using a sterile plastic pestle. Proteinase K (20 µl) and 4 µl of RNase was immediately added after homogenization, and samples were then digested for ~24 h in a 56 °C water bath. Samples were then vortexed for 15 s, 200 µl of AL buffer was added, and then incubated in a 70 °C water bath for 10 min. Following incubation, samples were vortexed for 15 s, and 200 µl of 96% EtOH was added. The mixture was then pipetted onto the spin column, and the DNA was bound. The columns were washed with AW1 buffer and then washed twice with AW2 buffer to further remove salts. Finally, total DNA was eluted with 200 µl of AE buffer. All the DNA samples were stored at −20 °C until quantification and further use.

### Quantification of *Wolbachia*

To obtain absolute quantification of *Wolbachia* in each individual bed bug, we used a droplet digital PCR (ddPCR^™^) system (Model QX200, Bio-Rad Laboratories, Hercules, CA, USA). The *Wolbachia*-specific primers, adopted from Sakamoto *et al*.^16^, targeted a region of the *Wolbachia* 16S gene that produced a 136 bp amplicon. We used primers for a ribosomal protein (RPL18) specific to *C. lectularius* as the reference gene due to its stability^56^; it produced a 137 bp amplicon. We used double-quenched TaqMan probes with a 5′ FAM fluorophore for *Wolbachia*, a 5′ HEX fluorophore for *C. lectularius*, and 3′ Iowa Black FQ quenchers with internal ZEN quenchers (Integrated DNA Technologies, Inc., Coralville, IA, USA) specific to each target. Primer and probe sequences are listed in Table 1. Template DNA was combined with *Wolbachia*-specific forward and reverse primers, TaqMan probes, and the ddPCR Supermix for Probes (Bio-Rad) into PCR-ready samples. The ddPCR reaction was optimized using extracted bed bug DNA that contained *Wolbachia*, and bed bug DNA that contained no *Wolbachia*, obtained from an established bed bug colony treated with antibiotics. The *Wolbachia*-free colony was fed weekly on defibrinated rabbit blood supplemented with rifampicin (10 µg/ml blood) and the Kao and Michayluk B Vitamin Solution (10 µl/ml blood) (Sigma-Aldrich, St. Louis, MO, USA) as adapted from Hosokawa *et al*.^8^. Repeatability of the ddPCR was assessed for detection of *Wolbachia* DNA and bed bug DNA on five different days and different experiments, and mean copy number (±SE) was used to calculate the coefficient of variation. Conventional PCR was used to verify amplification and respective band size with a 25 µl reaction of 12.5 µl GoTaq^®^ Green 2x Master Mix (Promega, Madison, WI, USA), 2.5 µl of template DNA, and 2.5 µl of each primer set (Table 1) under the following protocol: 95 °C for 2 min and (95 °C for 30 s, 60 °C for 30 s, 72 °C for 1 min) x 36 cycles, and 72 °C for 5 min.

**Table 1:** *Wolbachia*-specific (*w*Cle) and *Cimex lectularius* (*Clec*) reference gene primer set and TaqMan probe sequences used in PCR and ddPCR assays.

Primer/Probe	Sequence (5′-3′)
INTF2	AGTCATCATGGCCTTTATGGA
INTR2	TCATGTACTCGAGTTGCAGAGT
*w*Cle Probe	TGGTGTCTACAATGGGCTGCAAGG
RPL18F	GTATGACGGAGGCAGCTAGG
RPL18R	AACATTCGAGCAAATTCGGTA
*Clec* Probe	ATGAGGACGGTGTTCTTGCCTGTC

The bed bug/*Wolbachia* ddPCR assay comprised 22 µl of 1 × Droplet Supermix (Bio-Rad), 5 µl of bed bug template DNA, 2 U of *Mse*I restriction enzyme (New England Biolabs, Ipswich, MA, USA), 500 nM each of forward and reverse primers and 250 nM each of FAM- or HEX-labeled TaqMan probes for bed bug and *Wolbachia* strains, respectively. The 22 µl of PCR mixture was partitioned into an emulsion of ~20,000 droplets using the ddPCR system. PCR was performed on a T100 Thermal Cycler using the following protocol: 95 °C for 10 min and (94 °C for 30 s, 56 °C for 2 min) x 40 cycles, and 98 °C for 10 min. Post PCR, droplets were analyzed on the QX200 Droplet Reader. Absolute DNA amounts of bed bug and *Wolbachia* sequences in a sample were calculated on the Poisson distribution using the Quantasoft software version 1.7.4 (Bio-Rad). Data are reported as *Wolbachia* DNA (16S) copy number per individual bed bug and as *Wolbachia* DNA copy number per *C. lectularius* DNA (RPL18) copy number. Bed bug DNA samples containing *Wolbachia* (+control) and without *Wolbachia* (- control) were included in each experiment as checks on the ddPCR results. A no-template control was also included in each experiment to control for non-specific amplifications. To estimate the limit of detection of the ddPCR assay, serial dilutions of a DNA sample were prepared in water (5-, 25-, 125-, 625-, 3125-, and 15625-fold dilutions) and repeated three times. The limit of detection was estimated to be a single copy of either bed bug or *Wolbachia* target.

### Statistical analysis

We used a General Linear Model (GLM) Univariate Analysis of Variance (ANOVA) and Tukey’s HSD (*α* = 0.05) in SAS 9.4^57^ to test for differences in *Wolbachia* DNA per bed bug, the ratio of *Wolbachia* DNA to bed bug DNA across all life stages by bed bug strain, and between the two strains at each respective physiological stage. General linear models with factorial effects for strain and stage were fit to both the *Wolbachia* DNA and the ratio of *Wolbachia* DNA to bed bug DNA. Since residual plots indicated that both responses exhibited variability that was increasing with the mean, the log10-transformation was applied to both, residuals were checked for homogeneity of variance, and then analysis of variance (ANOVA) was carried out on the transformed data. Tukey’s HSD (α = 0.05) was used to test for differences in *Wolbachia* DNA per bed bug and also the ratio of *Wolbachia* DNA to bed bug DNA across all life stages for each fixed bed bug strain. Differences between the two strains at each respective physiological stage were also tested. Samples with low DNA yield (<15 copies/µl) of the bed bug reference gene RPL18 or *Wolbachia* 16S target were excluded from the analysis.

### Data availability

The data supporting the findings in this study are available as Supplementary Information.

## Electronic supplementary material

Supplementary Information
